# Customization of Computed Tomography Radio-Opacity in 3D-Printed Contrast-Injectable Tumor Phantoms

**DOI:** 10.3390/mi15080992

**Published:** 2024-07-31

**Authors:** Yuktesh Kalidindi, Aravinda Krishna Ganapathy, Liam Cunningham, Adriene Lovato, Brian Albers, Anup S. Shetty, David H. Ballard

**Affiliations:** 1 School of Medicine, Saint Louis University, St. Louis, MO 63104, USA; yuktesh.kalidindi@health.slu.edu; 2 School of Medicine, Washington University in St. Louis, St. Louis, MO 63110, USA; aganapathy@wustl.edu (A.K.G.); cunningham.l@wustl.edu (L.C.); 3 Mallinckrodt Institute of Radiology, Washington University School of Medicine, St. Louis, MO 63110, USA; lovato@wustl.edu (A.L.); anup.shetty@wustl.edu (A.S.S.); 4 St. Louis Children’s Hospital Medical 3D Printing Center, BJC HealthCare, St. Louis, MO 63110, USA; brian.albers@bjc.org

**Keywords:** 3D printing, attenuation, computer tomography, contrast, medical imaging phantoms

## Abstract

Medical Imaging Phantoms (MIPs) calibrate imaging devices, train medical professionals, and can help procedural planning. Traditional MIPs are costly and limited in customization. Additive manufacturing allows for customizable, patient-specific phantoms. This study examines the CT attenuation characteristics of contrast-injectable, chambered 3D-printed phantoms to optimize tissue-mimicking capabilities. A MIP was constructed from a CT of a complex pelvic tumor near the iliac bifurcation. A 3D reconstruction of these structures composed of three chambers (aorta, inferior vena cava, tumor) with ports for contrast injection was 3D printed. Desired attenuations were 200 HU (arterial I), 150 HU (venous I), 40 HU (tumor I), 150 HU (arterial II), 90 HU (venous II), and 400 HU (tumor II). Solutions of Optiray 350 and water were injected, and the phantom was scanned on CT. Attenuations were measured using ROIs. Mean attenuation for the six phases was as follows: 37.49 HU for tumor I, 200.50 HU for venous I, 227.92 HU for arterial I, 326.20 HU for tumor II, 91.32 HU for venous II, and 132.08 HU for arterial II. Although the percent differences between observed and goal attenuation were high, the observed relative HU differences between phases were similar to goal HU differences. The observed attenuations reflected the relative concentrations of contrast solutions used, exhibiting a strong positive correlation with contrast concentration. The contrast-injectable tumor phantom exhibited a useful physiologic range of attenuation values, enabling the modification of tissue-mimicking 3D-printed phantoms even after the manufacturing process.

## 1. Introduction

Medical Imaging Phantoms (MIPs) are specially designed models used to act as proxies for human tissues, playing a crucial role in calibrating and validating medical imaging devices and in the training of medical professionals [1]. Traditionally, MIPs were made using industrial techniques such as casting and molding, which limited the options to make patient-specific phantoms as they were often mass-produced. With the advent and improvement of 3D printing technology and increased accessibility, a transition has been made to developing 3D-printed MIPs. Along with this have come many benefits, including the ability to quickly produce patient-specific phantoms using previous imaging studies, change the properties of different parts of the MIP to imitate those of human tissues, and incorporate contrast agents [2,3,4].

Recent studies have demonstrated the utility of 3D-printed phantoms in various medical imaging applications. 3D-printed phantoms have been used in radiation therapy planning, dosimetry, evaluating the performance of computed tomography (CT) scanners, and developing dedicated nuclear medicine protocols and geometries [5,6,7,8]. The findings of these studies underscore the potential of 3D printing to enhance the customization and functionality of MIPs. Despite numerous studies, a gap in the literature presents itself in the use of contrast with MIPs, with most studies focusing on developing standardized tools to evaluate imaging modalities, compared to the application of modifying contrast concentration to mimic the physiologic processes as seen in our study. One such study by Driscoll et al. highlights the development of a dynamic flow imaging phantom using dynamic contrast-enhanced CT to compare imaging protocols [9].

Due to the high level of customization that comes with 3D-printed materials, a field of study has emerged to understand the possible applications of these materials in conjunction with contrast agents to improve visualization of sub-structures and mimic pathophysiologic processes [10,11]. In this study, the CT attenuation characteristics of contrast-injectable, chambered 3D-printed phantoms were assessed to aid in the construction of custom tissue-mimicking phantoms.

Many additive manufacturing techniques can be used in the fabrication and development of MIPs. Fused deposition modeling (FDM), stereolithography (SLA), and material jetting are three commonly utilized methods of 3D printing for biomedical applications [12,13,14]. FDM printing is carried out by sequentially extruding heated filament into a series of progressively layered two-dimensional patterns to construct a three-dimensional object. FDM is often the least complex and most cost-effective 3D printing method out of the three aforementioned techniques [15]. However, limitations in print resolution inhibit the accuracy with which FDM models can mimic complex anatomical structures [15,16]. Chances for leakage across the tumor phantom chambers also increase with FDM printing due to gaps that commonly form between adjacent extruded layers of the model [17]. SLA and material jetting, on the other hand, often utilize photopolymer resins that are cured upon exposure to UV light. During SLA printing, UV light is projected repeatedly into a vat of photopolymer resin until each layer of a model is cured on top of the next [15]. In mixed jetting printing, droplets of the material in use are deposited in the pattern of a model layer and sequentially cured with UV light [12]. Both techniques offer exceptional printing accuracy and resolution [15,18]. The curing process also chemically bonds each layer of photopolymer resin to the next, creating a less porous print that can contain liquid more effectively than an FDM model [19]. SLA printing is also more cost-effective than mixed jetting [20]. Thus, SLA printing was utilized for this study due to its high resolution, which is ideal for the intricacies of anatomical structures, and superior fluid-containing capabilities.

Tough 1500 resin was implemented for phantom material due to its strength and pliability, as well as its consistent radiopacity, with an average attenuation value of 101.4 HU, closely mimicking soft tissues, making it suitable for this application [21,22]. A preliminary model was printed in BioMed Durable resin. However, more translucent resins, such as BioMed Durable resin, tend to cause complications when printing hollow parts. The curing light from an SLA printer can pass through a clear section of the print and over-cure resin behind it [19]. This phenomenon can result in the sealing of smaller hollow structures like small blood vessels within the tumor phantom. The opaque nature of Tough 1500 made it a superior alternative to BioMed Durable resin.

**Figure 1:**
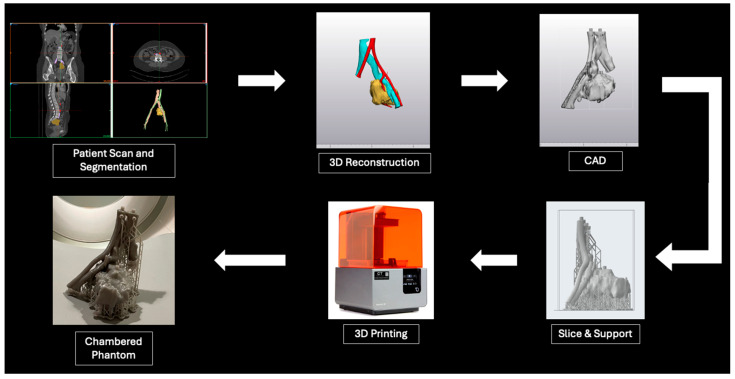
Illustration of the chambered 3D-printed tumor phantom manufacturing process.

A challenge currently faced by this emerging field that has not been fully addressed in the current research is the variability in contrast absorption and dispersion that occurs with different 3D-printed materials and techniques. In this study, chambered tumor phantoms were printed and injected with varying contrast solutions. By improving the understanding of contrast-injectable 3D-printed phantoms, our study aims to demonstrate the versatility of 3D printing in mimicking physiologic tissues and contrast distribution. These prints can be designed to enable contrast injection to specific parts of a phantom. Contrast concentrations in the injected solution can also be varied to achieve predetermined attenuation characteristics, further enhancing the tissue-mimicking and optimization capabilities of 3D-printed phantoms. The first set of goal attenuations for the tumor phantom was chosen by determining the attenuations exhibited by soft tissues (40 HU) and contrast-infused blood (150–200 HU) based on current literature [23,24]. The second set of goal attenuations was chosen based on attenuation ranges not represented by the first set of tumor phantom phases (90–150 HU) and common attenuation of low-density bone (400 HU) [25,26]. An illustration of the workflow to manufacture a chambered contrast-injectable 3D-printed phantom is shown briefly in Figure 1.

## 2. Materials and Methods

### 2.1. Tumor Phantom Construction

**Figure 2:**
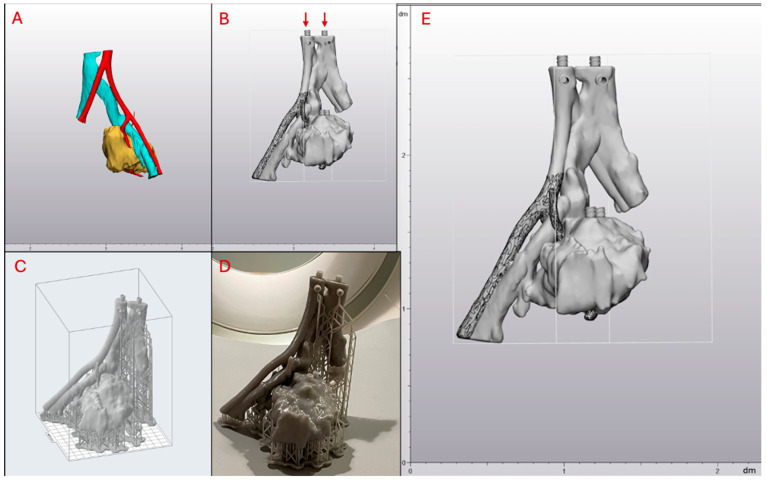
Depiction of the tumor phantom. (**A**) 3D reconstruction with three separate models. (**B**) CAD model with Luer Locks indicated by red arrows. (**C**) STL model in Preform 3.34.2 slicer software with supports. (**D**) Final 3D-printed tumor phantom on CT gantry. (**E**) CAD model of tumor phantom with rulers visible in decimeters (dm).

A CT scan of a patient with a complex pelvic chondrosarcoma close to the iliac bifurcation was obtained. The patient had a 3D-printed model used in their surgical planning, which is outside the purpose of this report. The CT scan was segmented to only include structures of interest, such as the distal abdominal aorta and inferior vena cava, proximal iliac arteries and veins, and the tumor itself. These desired anatomical structures were segmented separately, ensuring each part was its own model, as depicted in Figure 2A. The volumes of each anatomical structure are outlined in Table 1. These models were then imported into a CAD program (3-matic), where they were cleaned, repaired, and smoothed as necessary. Each of the three individual anatomical parts was given a 2 mm offset, and the original model was subtracted from the inside of the larger offset piece to create a hollow part. The models were inspected for overlaps that could cause leaks between different parts and were repaired as needed to ensure the chambers remained separated. Luer lock connectors were 3D modeled and added to the models as required, with holes created to allow for injection, as shown in Figure 2B. All three parts were combined within the CAD program to create a single piece from the assembly. The final CAD model had dimensions of 11.9 × 12.5 × 18.7 cm and is depicted with rulers visible in Figure 2E.

The combined model was then imported as an STL file into PreForm 3.34.2 (Formlabs Inc., Somerville, MA, USA), a slicing software specifically for 3D printers manufactured by Formlabs (Formlabs Inc., Somerville, MA, USA). PreFrom contains predetermined print settings for each Formlabs resin, with layer thickness being the only modifiable print parameter. Once Tough 1500 resin was selected as the print material and layer thickness was set to 0.100 mm, supports were generated.

First, only external supports were added to the model using the auto-generate feature in PreFrom. The slicing software constructed these external supports automatically without user intervention. Internal supports were added manually. The tumor phantom needed to be printed in a manner that minimized internal supports inside each sealed chamber since internal support removal after printing would be impossible. Printing with no internal supports at all, however, would significantly increase the chances of print failure due to the structural complexity of the model. The ideal tumor phantom orientation in PreForm was found by repeatedly changing orientation and clicking auto-generate supports. The phantom was then fixed at the orientation with the least amount of internal support. Care was also taken to ensure this orientation had the topmost injection ports vertically positioned, as shown by the red arrows in Figure 2B. All internal supports were removed, and a smaller amount was manually added back only in regions characterized by the slicing software as “unsupported minima”, which are high-risk portions of the model that can lead to print failure. The external support point size was set to 0.5 mm with a density of 0.8. The manual supports added to the inside of the model had a point size of 0.6 mm.

**Figure 3:**
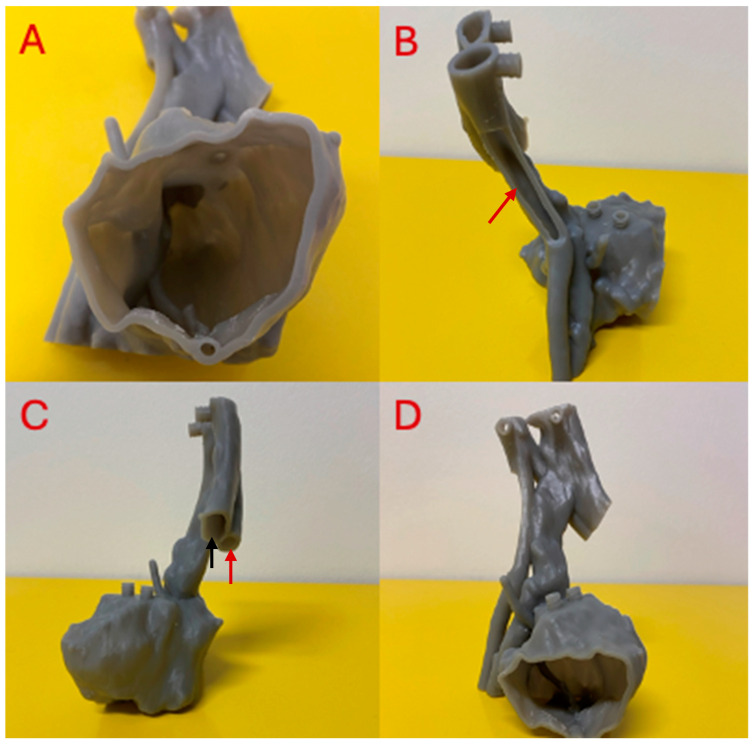
Depiction of the inside of all tumor phantom hollow chambers after support removal. This version of the tumor phantom provides views of (**A**) the tumor chamber interior, (**B**) the arterial chamber interior indicated by red arrows, and (**C**) the venous chamber interior indicated by the black arrow. (**D**) Axial view of the chamber-exposed version of the 3D-printed tumor phantom.

The phantom was then printed on a Formlabs Form 3 3D printer (Formlabs Inc., Somerville, MA, USA) using Tough 1500 resin. The final tumor phantom had 1768 layers and took 13 h to print. The volume of 1500 resin utilized was 140 mL. For post-processing, luer lock syringes filled with 99% isopropyl alcohol (ISP) were used to flush the internal cavities of the phantom thoroughly. The phantom was filled to capacity and emptied twice. The print was then placed in a Form Wash L (Formlabs Inc., Somerville, MA, USA) agitation tank filled with 99% ISP to clean the model’s exterior. Two wash cycles of 10 min each were conducted. The model was dried in a dehydrator for two hours to remove all remaining ISP and then cured in the Form Cure L (Formlabs Inc., Somerville, MA, USA) curing oven for 60 min at 70 degrees Celsius. All wash and curing protocols were carried out as recommended by the Tough 1500 printing guidelines from Formlabs [27,28]. External supports were not removed before scanning in order to ensure the phantom was able to be situated upright on the CT gantry, as depicted in Figure 2D. A second version of the 3D-printed tumor phantom with each hollow chamber exposed was printed to provide an internal view, as shown in Figure 3.

### 2.2. Tumor Phantom Contrast Injection

The desired attenuation values for arterial phase I, venous phase I, and tumor phase I scans were the following: 200 HU, 150 HU, and 40 HU. First, five separate contrast solutions were made to determine the ideal *v*/*v* percentage of iodinated contrast in water to yield a tumor phantom attenuation of 40 HU. Each solution contained 0.1 cc to 0.5 cc of Optiray 350 iodinated (Guerbet LLC, Princeton, NJ, USA) contrast and 80 cc of water. One of these solutions was injected into the tumor chamber till capacity was reached, and the phantom was scanned on a Siemens (Erlangen, Germany) Biograph CT scanner at a current of 100 mAs and voltage of 120 kVp. All CT analysis and segmentation were performed using Materialise Mimics 26.0 (Leuven, Belgium) image processing software. For each scan, two study investigators placed one region of interest (ROI) on the contrast-filled tumor portion of the phantom in each one of the three CT planes. The placement of each ROI was left up to the discretion of each investigator. Each ROI was ensured to have an area of 40–50 mm^2^. The attenuation value of the tumor phase was acquired by averaging the mean attenuation of all six circular ROIs. This process was repeated with the other contrast solutions until 0.4 cc of Optiray contrast in 80 cc of water was determined to be the desired concentration to yield a tumor chamber attenuation value of around 40 HU. The contrast solution concentrations required for the other two phases were determined by assuming a linear relationship between contrast concentration and attenuation and extrapolating the tumor phase concentration:

$$Volume\;of\;iodinated\;contrast\;in\;80\;mL\;water\;for\;200\;HU=200\;HU\times \frac{0.4\;cc}{40\;HU}=2\;cc\;contrast$$

$$Volume\;of\;iodinated\;contrast\;in\;80\;mL\;water\;for\;150\;HU=150\;HU\times \frac{0.4\;cc}{40\;HU}=1.5\;cc\;contrast$$

Since 200 HU was the desired attenuation for the arterial phase scan, 2 cc of contrast was mixed with 80 cc of water. For the venous phase scan, 1.5 cc of contrast was mixed with 80 cc of water. The volume/volume (*v*/*v*) percentage of these solutions is outlined in Table 2.

The injection and scanning process outlined for the tumor phase scan was repeated for the arterial and venous phase scans with their contrast solutions of predetermined concentrations. Only one chamber was injected at a time while ensuring the chamber was filled to capacity before each scan. The tumor phantom was scanned using the same current and voltage as the initial tumor phase scan. The phantom was flushed with water and emptied three times after each scan before injecting a different contrast solution into its respective chamber. Attenuation values for the arterial and venous phase scans were gathered in the same manner as the tumor phase scan. The only difference was the ROI placement on the artery and vein chambers of the phantom—to reflect arterial and venous phase scans, respectively. The attenuation values for each phase were then averaged and compared to the desired attenuation values by calculating percentage differences, as shown in Table 3.

Next, an additional set of three contrast solutions were injected into the tumor phantom and subsequently analyzed. Goal attenuation values for venous phase II, arterial phase II, and tumor phase II were chosen to be 90 HU, 150 HU, and 400 HU. The volumes of contrast required to achieve each phase II goal attenuation were calculated by utilizing results from phase I scans exhibited in Table 3. The observed attenuation of the venous phase I scan was 201.42 HU, as shown in Table 3, and achieved by mixing 1.5 cc of contrast in 80 cc of water. The required contrast volumes in 80 cc of water to achieve 90 HU, 150 HU, and 400 HU were calculated by using this ratio of approximately 1.5 cc per 200 HU:

$$Volume\;of\;iodinated\;contrast\;in\;80\;mL\;water\;for\;400\;HU=400\;HU\times \frac{1.5\;cc}{200\;HU}=3\;cc\;contrast$$

$$Volume\;of\;iodinated\;contrast\;in\;80\;mL\;water\;for\;90\;HU=90\;HU\times \frac{1.5\;cc}{200\;HU}=0.68\;cc\;contrast$$

$$Volume\;of\;iodinated\;contrast\;in\;80\;mL\;water\;for\;150\;HU=150\;HU\times \frac{1.5\;cc}{200\;HU}=1.13\;cc\;contrast$$

**Figure 4:**
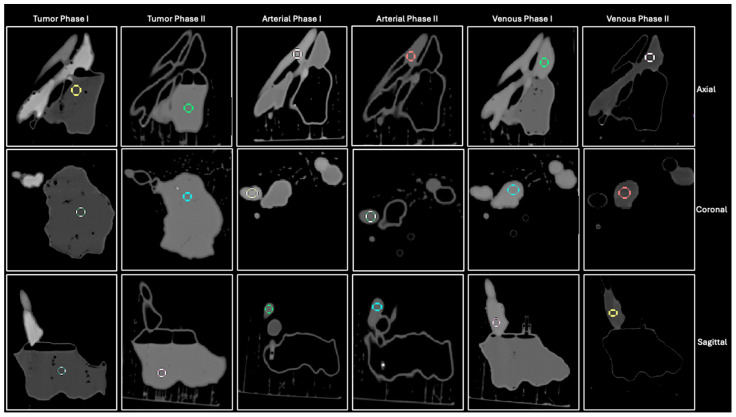
CT images of all six phases of tumor phantom in axial, coronal, and sagittal planes along with ROI placement by Rater 1 in each image depicted by colored circles.

**Figure 5:**
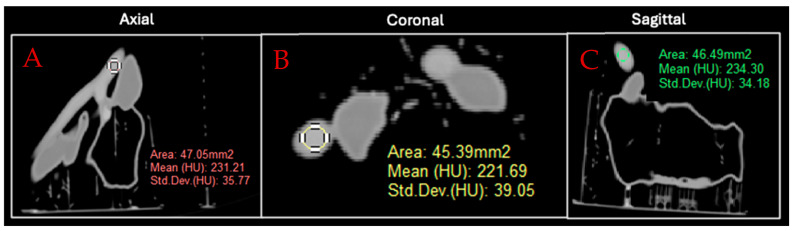
CT images of tumor phantom arterial phase I in (**A**) axial, (**B**) coronal, and (**C**) sagittal planes with ROI placement (colored circles) and depiction of attenuation measurement.

The final contrast volumes utilized, along with the *v*/*v* percentages of each solution, are outlined in Table 4. The aforementioned contrast injection, CT scanning, and image analyzation process were then repeated for the second set of three contrast solutions. CT images of all six phases in all three CT planes, along with ROI placement by rater 2, are depicted in Figure 4. Imaging parameters acquired from each ROI included mean attenuation, standard deviation, and ROI area, as outlined in Figure 5 for arterial phase I. The attenuation values for each phase were then averaged and compared to the desired attenuation values by calculating percentage differences, as shown in Table 3.

**Figure 6:**
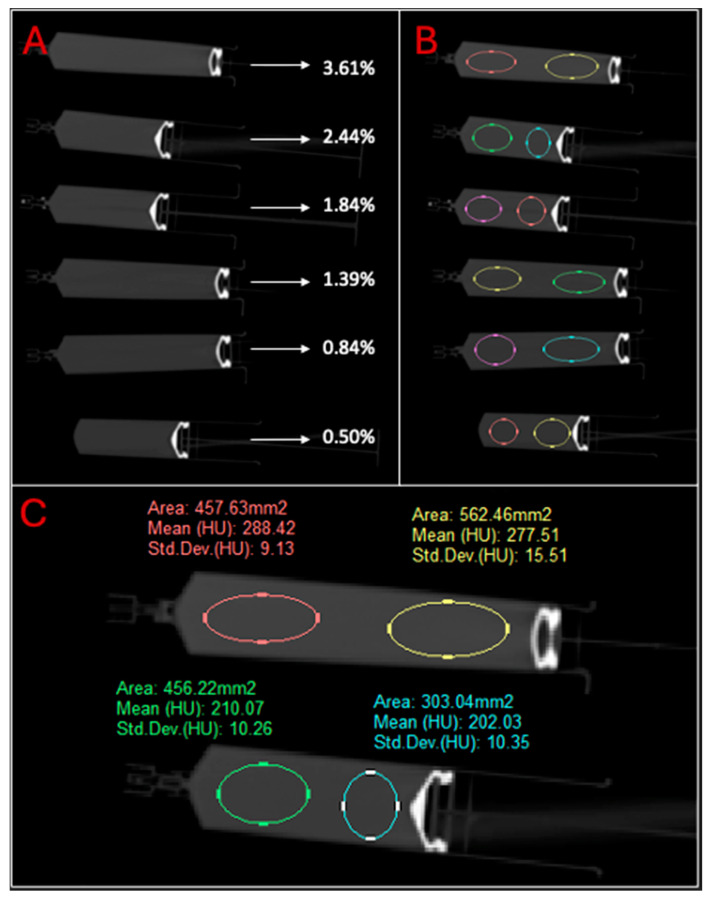
(**A**) Coronal CT image of contrast solution syringe gantry arrangement with concentration expressed as *v*/*v* percentage. (**B**) ROI placement within each syringe. (**C**) ROI placement for 3.61% and 2.44% contrast solution syringes shown with colored circles and corresponding labels with attenuation measurements.

**Figure 7:**
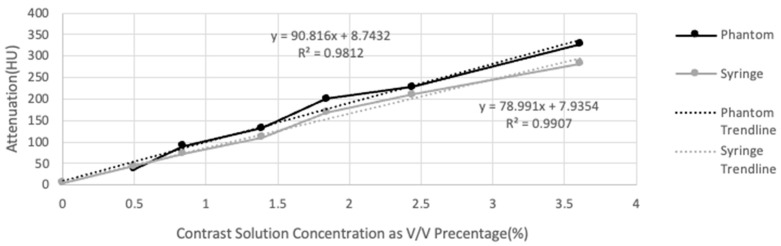
Tumor phantom and syringe attenuation (HU) graphed against contrast solution concentration utilized for each phase represented using volume/volume percentage (%).

Syringes containing all the contrast solutions used in this study were also scanned with CT, as depicted in Figure 6. Two study investigators each placed two elliptical ROIs on each syringe. ROI placement was left up to the discretion of each study investigator. Each ROI was ensured to have an area of 300–600 mm^2^ and placed entirely within the borders of each syringe. The mean attenuation for the contrast solution in each syringe was then calculated and reported in Table 5. The attenuations of each syringe and respective tumor phantom phase were charted against the utilized solution’s concentration in Figure 7.

Statistical analyses were conducted using Rstudio version 2024.04.2. Graphs and tables were created using Microsoft Excel version 16.77.1, which was accessed on 21 July 2024. Comparisons between tumor phantom and syringe attenuation values were performed using a two-tailed, paired *t*-test. Interrater analysis between the two CT readers for both tumor phantom and syringe attenuations was conducted by determining intraclass correlation coefficient (ICC) values. ICC values between 0.75 and 0.9 were taken to represent good reliability, whereas values greater than 0.9 indicated excellent reliability.

## 3. Results

### 3.1. Contrast Injection and Phantom Attenuation

All six phases of the 3D-printed tumor phantom were visible on CT, as depicted in Figure 4. Despite observable leakage between chambers on CT imaging, the injected chambers in each phase were filled to capacity. Tumor phase I exhibited a mean HU of 37.49 ± 3.95, venous phase I exhibited a HU of 200.50 ± 4.03, and arterial phase I exhibited a HU of 227.92 ± 5.21. Tumor phase II exhibited a mean HU of 326.20 ± 2.70, venous phase II exhibited a HU of 91.32 ± 1.90, and arterial phase II exhibited a HU of 132.08 ± 5.43 (Table 3).

As expected, the attenuation values reflected the relative concentrations of contrast solutions utilized for each phase. Tumor phase II was injected with the most concentrated contrast solution with a *v*/*v* percentage of 3.61% and exhibited the highest attenuation value (326.20 ± 2.70) out of the three phases. Tumor phase I was injected with the least concentrated solution at 0.50%, resulting in the lowest mean attenuation value at 37.49 ± 3.95. The other tumor phantom phases exhibited increasing observed mean attenuation as their respective injected contrast solution concentrations increased. Tumor phantom phases arranged in ascending order of their exhibited mean attenuation are outlined as follows: tumor I, venous II, arterial II, venous I, arterial I, and tumor II.

The percent differences between goal attenuation and observed attenuation were 6.48% for tumor phase I, 13.05% for arterial phase I, 28.82% for venous phase I, 20.32% for tumor phase II, 12.71% for arterial phase II, and 1.45% for venous phase II. Venous phase II had the lowest percentage difference, whereas venous phase I exhibited the highest. The mean percent difference decreased from 16.1% for the phase I scans to 11.5% for the phase II scans. Although percent differences were considerably high, the observed attenuation values were similar to goal attenuation values with regard to relative HU differences between phases. The difference between observed arterial I and venous phase I attenuation values was still considerably smaller (28 HU) than the difference between observed tumor I and arterial phase I values (191 HU) or tumor and venous phase I values (163 HU). Similarly, observed arterial II and venous phase II attenuations had a relatively low difference of 41 HU, while the attenuation differences between tumor phase II and arterial phase II (194 HU) or tumor phase II and venous phase II (235 HU) were relatively larger. For both sets of scans, the relative differences in observed mean attenuations reflect the attenuation differences between goal attenuation values.

### 3.2. Attenuation versus Concentration

Phantom mean attenuations for each concentration were consistently higher compared to syringe attenuations except for the tumor phase I solution (Figure 7). The difference in mean attenuations between the phantom and syringe datasets proved to be statistically significant upon analysis with a paired *t*-test at a significance threshold of 0.05 (*p*-value = 0.03137).

The observed mean attenuation of each phase increased for both the tumor phantom and syringes as contrast solution concentration increased (Table 5, Figure 7). R-values were 0.990 for tumor phantom attenuation and 0.995 for syringe attenuation, indicating a strong positive linear correlation between attenuation and concentration for both datasets. Phantom attenuation and syringe attenuation plotted against concentration yielded R-squared values of 0.981 and 0.991, which convey that changes in concentration are highly predictive of changes in attenuation. Trendlines for phantom attenuation versus concentration and syringe attenuation versus concentration exhibited slopes of 90.82 and 78.99, respectively.

**Figure 8:**
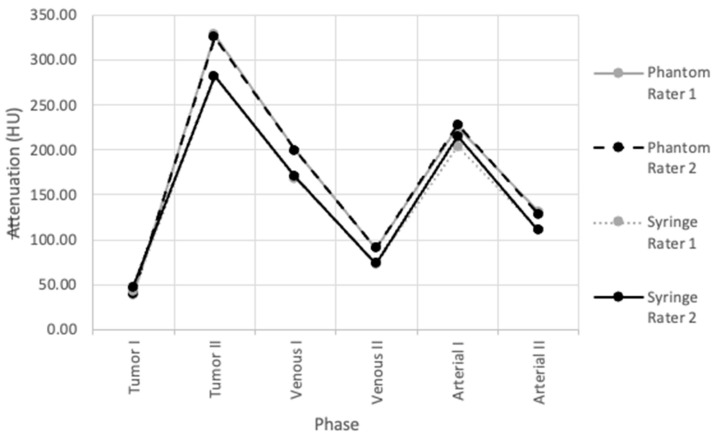
ICC interrater analysis of both readers for tumor phantom and syringe attenuations.

The variation in phantom and syringe attenuation data acquired by both readers was visualized by plotting the data collected by each investigator against each other for all six phases (Figure 8). The ICC values for tumor phantom and syringe attenuations were 0.9998415 and 0.9987372, respectively, which are within the ‘excellent’ reliability range (ICC = 0.90–1.00) (Table 6).

## 4. Discussion

The objective of this study was to understand the attenuation characteristics and customization capabilities of contrast-injectable, additively manufactured imaging phantoms. A major benefit of additive manufacturing imaging phantoms is the wide selection of available 3D printing materials with diverse radiological properties. This study aimed to expand upon this advantage by enabling the static, intrinsic attenuation characteristics of a 3D-printed material to be customizable via contrast injection. 

The final observed attenuations of each phase (tumor I: 37.49 HU, tumor II: 326.20 HU, venous I: 200.50 HU, venous II: 91.32 HU, arterial I: 227.92 HU, arterial II: 132.08 HU) were different from their respective goal attenuations (40 HU, 400 HU, 150 HU, 90 HU, 200 HU, 150 HU) with only venous phase II exhibiting a percent difference less than 5%. The relative differences in observed attenuation between phases proved to be similar to differences in goal attenuation. CT imaging often relies on the differences in attenuation values between tissues to enhance understanding of underlying pathology. For example, liver tissue is largely homogeneous in non-contrast CT. The triple-phase liver protocol is often used to differentiate between healthy liver parenchyma and a cancerous lesion. Some studies have shown a mean difference of around 26 HU between a liver lesion and the surrounding healthy parenchyma in arterial phase scans [23]. A 3D-printed phantom with sealed chambers can be injected with different contrast solutions into adjacent chambers to mimic pathologic lesions adjacent to normal physiologic tissue. The difference between the observed arterial and venous phase attenuations in this study was around 28 HU, proving contrast-injectable 3D-printed phantoms capable of reproducing attenuation differences adjacent physiologic tissues can exhibit on CT imaging. 

The absolute attenuation values of the tumor phantom proved to be within a useful physiologic attenuation range. The tumor phase I scan exhibited an attenuation of 37.49 HU, which is close to the attenuation values for blood (38.89 HU), renal parenchyma (renal cortex and medulla, 33.2 ± 4.4 HU and 34.2 ± 4.8 HU, respectively), spleen (37.6 HU), unenhanced liver parenchyma (right lobe of liver, 47.5 HU), and psoas muscles (44.0–44.4 HU) [29,30,31]. The attenuation values observed in the arterial I/II and venous phase I/II scans (201.42 HU, 91.32 HU, 229.3 HU, 132.08 HU) correlated closely with the arterial and venous phase aorta CT attenuation ranges noted in pancreatic cancer patients [32,33]. Hepatocellular carcinoma and contrast-enhanced liver parenchyma attenuation values during hepatic arterial and venous phases (80 HU to 120 HU, 60 HU to 130 HU) were similar to arterial and venous phase II scan attenuations [34]. The mean attenuation exhibited by tumor phase II of 326.20 HU also falls within the range of HU values for trabecular bone [35]. Thus, a single 3D-printed tumor phantom with contrast compatibility can represent a wide array of physiologic and pathologic tissues. 

The tumor phantom utilized in this study was constructed out of FormLabs Tough 1500 resin. This photopolymer has an average attenuation value of 101.4 HU [22]. Through the injection of solutions with varying iodinated contrast concentrations into the chambered phantom, the attenuation of the phantom was modified to have a range of 37.49–326.20 HU. A solid tumor phantom made of the same Tough 1500 material, on the other hand, could only represent tissues with an attenuation of 101.4 HU.

Phantom attenuation exhibited a strong positive correlation with contrast solution concentration, as depicted in Figure 7. An R-squared value of 0.986 also indicates that contrast concentration is a reliable predictor of attenuation even when contrast is injected into a chambered 3D-printed phantom. Although attenuation was consistently higher in the tumor phantom compared to the syringes, this statistically significant difference is likely attributable to inadequate agitation of solutions before scanning and CT artifact noted within syringes. The similarity in the rate of change of both phantom and syringe attenuation trendlines—90.82 and 78.99, respectively—confirms that contrast affected attenuation in a largely similar, predictable manner within both containers. The tumor phantom in this study exhibited a 90.82 HU increase in attenuation for every 1% increase in concentration. Consequently, the trends gathered from the attenuation values in this study can be utilized to make contrast solutions that can achieve a predetermined HU value within the 3D-printed phantom. Once the change in a specific 3D-printed phantom’s attenuation relative to contrast concentration is determined, its tissue-mimicking capabilities can be enhanced significantly by simply varying the composition of the solution injected.

This study is not without limitations. Leakage between chambers was observed, which could have influenced the accuracy of the attenuation measurements. Additionally, the range of contrast concentrations tested was limited, and only one type of 3D printing resin was utilized. Attempts to conduct a dimensional analysis were also limited by the lack of clear borders when segmenting the contrast-filled tumor phantom cavities. Future studies should address these limitations by using larger sample sizes, improving the sealing of chambers, and exploring a broader range of contrast concentrations and materials. More robust methods for a dimensional comparison between the tumor phantom and the segmented patient anatomy should also be explored.

## 5. Conclusions

3D-printed phantoms can be designed to enable contrast injection into a specific chamber. The contrast-injectable tumor phantom in this study exhibited a useful physiologic range of attenuation values, which enables the modification of 3D-printed MIPs even after the manufacturing process. Contrast concentrations in the injected solution can also be varied to achieve predetermined attenuation characteristics, further enhancing the tissue-mimicking and optimization capabilities of 3D-printed phantoms.

## Figures and Tables

**Table 1:** Volumes of each anatomical structure within segmented 3D reconstructions of the patient CT scan.

Anatomical Structure	Volume (mL)
Artery	12.96
Vein	29.18
Tumor	160.44

**Table 2:** Contrast volume in 80 mL water and *v*/*v* percentages of each solution utilized in phase tumor I, venous I, and arterial I.

Phase	Goal Attenuation (HU)	Volume of Contrast in 80 mL Water (mL)	Volume/Volume Percentage
Tumor I	40	0.4	0.50%
Venous I	150	1.5	1.84%
Arterial I	200	2	2.44%

**Table 3:** Percent differences between goal and observed mean attenuation values of all six tumor phantom phases.

Phase	Goal Attenuation (HU)	Observed Mean Attenuation (HU)	Percent Difference
Tumor I	40	37.49 ± 3.95	6.48%
Tumor II	400	326.20 ± 2.70	20.32%
Venous I	150	200.50 ± 4.03	28.82%
Venous II	90	91.32 ± 1.90	1.45%
Arterial I	200	227.92 ± 5.21	13.05%
Arterial II	150	132.08 ± 5.43	12.71%

**Table 4:** Contrast volume in 80 mL water and *v*/*v* percentages of each solution utilized in phase tumor II, venous II, and arterial II.

Phase	Goal Attenuation (HU)	Volume of Contrast in 80 mL Water (mL)	Volume/Volume Percentage
Tumor II	400	3	3.61%
Venous II	90	0.68	0.84%
Arterial II	150	1.13	2.44%

**Table 5:** Syringe contrast solution concentrations expressed as *v*/*v* percentage and mean attenuation of each solution.

*v*/*v* Percentage (%)	Mean Attenuation (HU)
0.5	44.69 ± 4.26
0.84	73.01 ± 5.00
1.39	110.99 ± 3.81
1.84	169.19 ± 3.47
2.44	210.12 ± 8.24
3.61	281.87 ± 6.02

**Table 6:** ICC values quantifying interrater analysis of both readers for tumor phantom and syringe attenuations.

Attenuation Type	ICC Value
Tumor phantom	0.9998415
Syringe	0.9987372

## Data Availability

The data presented in this study are available in the article.
